# The Changed Probationer

**Published:** 1920-05-15

**Authors:** 


### THE MATRONS' AND SISTERS' DEPARTMENT.

### THE CHANGED PROBATIONER.

The legacy which Florence Nightingale left to the sisters she trained was to trust their own generation. It is something like treachery to her principles when sisters lament that probationers are not what they used to be. As a matter of fact, they never were. The very sisters who now expatiate on the degeneration of candidates for training were themselves the subject of the same complaint when they first began work. For every generation displays different characteristics. The younger women, particularly, present an astonishing variety of type even from one decade to another. They hold their ideals under diverse forms and are perennially impatient at the expression of those which inspired their elders. It is hard sometimes to realise that under these changing surface manifestations they come of the good old stock and may be depended on to carry the banner of nursing efficiency forward into the future. Yet so it is.

At the same time it ought to be kept in mind that training-schools have changed considerably in one important circumstance during the last few years. They used to be composed entirely of women over twenty-three. Many of the senior probationers in days gone by were nearing thirty. Their outlook on the world was mature. They knew what they wanted in life and had decided deliberately, often after making trial of some other avocation, that the career of a nurse was the best thing. We have lowered the age at which women begin to enter the profession, and some are in danger of forgetting that the change involves other alterations.

Superintendents of nurses in America are beginning to discover that to govern a household composed of women just emerged from childhood entails a rule different in many particulars from that which was needed by women commencing their apprenticeship to nursing four years later. The modern girl of nineteen is fully as independent, fully as capable of taking care of herself, perhaps, as the girl of twenty-three used to be. But the gulf which divides the two ages is one which is unaffected by changing conventions, tastes, and fashions. Between the age of nineteen and that of twenty-three lies a seething period of development. When the training-schools decided to invite candidates to spend these years in the study and practice of nursing they conferred a priceless boon on women traversing this receptive period of life, but they undertook responsibilities to match.

Many lives are thrown away at this period, many promising careers wasted for lack of that collegiate, as opposed to conventual, discipline and restraint which enable the young to protect themselves against the most obvious dangers of adolescence.

Unless the young probationer find interests set before her which will widen her outlook on life during these impressionable years, she will fall a prey to love of excitement, squander her scanty means in the hunt for pleasure, and leave her training-school coarsened and narrowed instead of ripened.

In commemorating, as we do this week, the centenary of Florence Nightingale's birth, it is wholesome to reflect on her attitude towards the pupils at the Nightingale School. It was her doctrine that the training of nurses involves the development of all their faculties; that the complete nurse is a woman finely endowed whose opportunities for usefulness have brought her to a higher comprehension of what life means for herself, and that no trouble is thrown away which is spent in fitting the nurse in training for a balanced and influential career.

The art of influence does not consist in admonitions, in surveillance or the setting up of barriers. To be of any avail it must fall obliquely and enter the heart, not the head. It consists in great part of providing opportunities. It encourages enterprise, leaves the planning in the hands of those most nearly concerned, and assigns to authority the rôle of the goddess out of the machine, to remove difficulties and achieve the impossible when other resources are exhausted. The probationer where this impalpable influence in the direction of higher things is in the air finds herself caught in the swirl of the movement before she has been many hours in residence. She is called upon by her fellows to take her share, to contribute her talents, to *do* something.

If we are to have these young probationers brimming over with life, only half-tamed as it were, let us take heed that we fill their leisure with interests which will advance their bodily, their mental, their spiritual welfare.

